# Raloxifene in Hemodialysis Patients

**DOI:** 10.5812/ijem.5457

**Published:** 2012-06-30

**Authors:** Frieder Keller

**Affiliations:** 1 Nephrology Department, University Hospital, Germany

**Keywords:** Raloxifene, Oxidative Stress

Dear Editor,

There is good news for the renal patients since more treatment options have been derived for Chronic Kidney Disease-Mineral Bone Disorder (CKD-MBD) apart from suppressing parathyroid hormone levels. The role of parathyroid hormone apparently seems to be clear. However, it is still unclear what ‘adynamic bone’ disease really means.

Whether renal patients treated with supra-physiological parathyroid hormone would not suffer from adynamic bone disease? Or ‘adynamic bone disease’ is a specific form of osteoporosis in renal patients?

The beneficial effect of raloxifene, an established osteoporosis treatment now has been shown on bone mineral density in both, diabetic and non-diabetic dialysis patients (1). Bone resorption was less, parathyroid hormone levels decreased and bone density was improved by raloxifene. Bone mineral density also improved while on raloxifene treatment in postmenopausal women with CKD before dialysis (2). Thus, ‘adynamic bone disease’ the presumed osteoporosis subtype of CKD-MBD could be seen as the metabolic consequence of sex hormone deficits. The next step might be a raloxifene trial even in men with CKD. At present it will be prudent, however, to use the lower dose of 60 mg per day since raloxifene exposure is higher in renal patients (3). In addition, such treatment should be restricted to 1 year because safety and cardiovascular risks after long-term use are still unknown in this population.
